# Infection of novel reassortant H1N2 and H3N2 swine influenza A viruses in the guinea pig model

**DOI:** 10.1186/s13567-018-0572-4

**Published:** 2018-07-27

**Authors:** Rodrigo Tapia, Victoria García, Juan Mena, Sergio Bucarey, Rafael A. Medina, Víctor Neira

**Affiliations:** 1 Departamento de Medicina Preventiva Animal, Facultad de Ciencias Veterinarias y Pecuarias, Universidad de Chile, Santa Rosa, 11735 Santiago, Chile; 2 Programa de Doctorado en Ciencias Silvoagropecuarias y Veterinarias, Universidad de Chile, Santa Rosa, 11735 Santiago, Chile; 3 Departamento de Enfermedades Infecciosas e Inmunología Pediátrica, Escuela de Medicina, Pontificia Universidad Católica de Chile, Santiago, Chile; 4 Department of Microbiology, Icahn School of Medicine at Mount Sinai, New York City, NY USA; 5 Millennium Institute on Immunology and Immunotherapy, 391 Marcoleta, Santiago, Chile

## Abstract

Novel H1N2 and H3N2 swine influenza A viruses (IAVs) were identified in commercial farms in Chile. These viruses contained H1, H3 and N2 sequences, genetically divergent from IAVs described worldwide, associated with pandemic internal genes. Guinea pigs were used as human surrogate to evaluate the infection dynamics of these reassortant viruses, compared with a pandemic H1N1 virus. All viruses replicated and were shed in the upper respiratory tract without prior adaptation although H1N2 viruses showed the highest shedding titers. This could have public health importance, emphasizing the need to carry out further studies to evaluate the zoonotic potential of these viruses.

**Electronic supplementary material:**

The online version of this article (10.1186/s13567-018-0572-4) contains supplementary material, which is available to authorized users.

## Introduction, methods and results

Influenza A virus (IAV) has economic and public health relevance, being considered ubiquitous in the swine industry worldwide [1, 2]. There was limited information about IAVs circulating in swine population around the world, but the pandemic H1N1 (pdmH1N1) lineage that emerged in 2009 underscored the need to increase the surveillance and research of IAV in swine (IAV-S) [3]. In Chile, novel IAVs-S of the H1 and H3 subtypes have been recently identified in commercial swine farms, which are genetically divergent from IAVs described in other countries. These novel IAV-S lineages were most closely related to human seasonal H1N1 and H3N2 viruses from the late 1980s and early 1990s and were named: Chile H1 human I, Chile H1 human II, Chile H3 human I, and Chile H3 human II [4]. To date, phylogenetic analyses have been based on hemagglutinin (HA) sequences only, highlighting the need for additional studies to fully characterize these viruses, including the evaluation of their zoonotic potential.

Different animal models have been used for the study of IAVs, with guinea pigs, ferrets and mice being the most frequently used. Guinea pigs have several advantages compared to ferrets, such as their commercial availability, small size, low cost, and their ease of handling and housing, which make them an attractive model for IAV. In addition, they have been shown to have a high susceptibility to low-passage human isolates, in contrast to mice, in which virus adaptation is usually required for efficient infection [5, 6]. Guinea pigs have been used to assess the 2009 pdmH1N1 [7, 8] and seasonal H1N1 and H3N2 human influenza viruses [9, 10], because the anatomy and physiology of the guinea pig respiratory system resembles that of humans [11]. However, few studies have used this animal model to assess novel IAVs-S circulating on commercial farms worldwide, which may pose a risk to human public health. Hence, we evaluated the replication kinetics and shedding of novel reassortant H1N2 and H3N2 IAVs-S in the guinea pig model, compared with a pdmH1N1 virus.

We isolated pdmH1N1, H1N2 and H3N2 IAVs-S during passive and active surveillance carried out on Chilean commercial swine farms in 2014–2015. Thirty-two farms belonging to 22 companies, which represent >90% of the pig inventory in Chile, were enrolled in the study. The farms were integrated one-site or multi-site farming systems with greater than 800 sows in the farrowing units.

Briefly, TRIzol^®^ LS Reagent (Invitrogen™, Carlsbad, CA, USA) was used to isolate RNA from nasal swab or oral fluid samples, and a real time RT-PCR based on highly conserved regions of the IAV matrix (M) gene was carried out [12]. Positive RT-PCR samples were inoculated in Madin-Darby Canine Kidney (MDCK) cell monolayers and were incubated for 1 h at 37 °C to allow virus absorption. Cells were then rinsed with 1× phosphate-buffered saline (PBS) to remove unbound virus, and IAV growth medium (MEM supplemented with 1 μg/mL of trypsin treated with *N*-tosyl-l-phenylalanyl chloromethyl ketone (TPCK), 0.3% bovine serum albumin, and 1% antibiotic–antimycotic solution) was added. The monolayers were incubated at 37 °C and observed for cytopathic effect (CPE) daily for 5 days.

The HA gene sequences of isolated IAVs-S were obtained by Sanger sequencing, which was performed at Veterinary Diagnostic Laboratory, University of Minnesota, Saint Paul, MN, USA. Sequence alignment of the HA gene was carried out with MUSCLE and phylogenetic trees were constructed with the Maximum likelihood method and a General Time Reversible model with a variation rate among sites given by gamma distribution with invariant sites (GTR + G+I), using MEGA v7.0 software. Reference sequences of swine and human influenza viruses were selected from GenBank [13], and from searches using BLAST [14] and the Influenza Research Database [15]. Bootstrap values were determined with 1000 replicates of the dataset. The new swine H1 clade classification tool was used to determine the identity of H1 viruses [15, 16].

**Figure 1:**
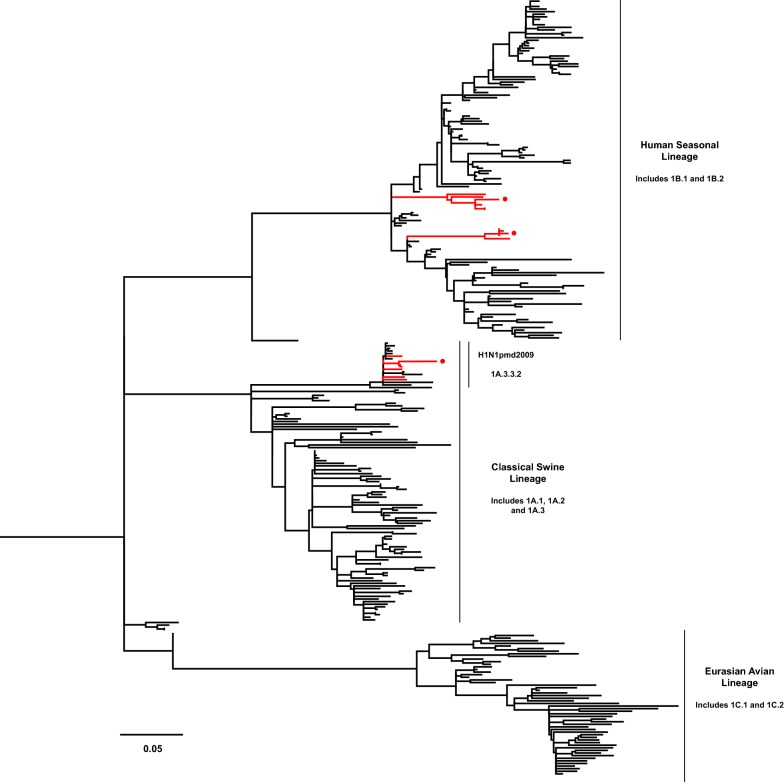
**Phylogenetic trees based on HA sequences of subtype H1.** Maximum Likelihood method and General Time Reversible model with a variation rate among sites given by gamma distribution with invariant sites (GTR + G + I) were used. Human and swine reference sequences are in black. Branches of Chilean IAVs-S sequences are in red, and nodes of selected viruses for guinea pig infection are highlighted with a red circle.

**Figure 2:**
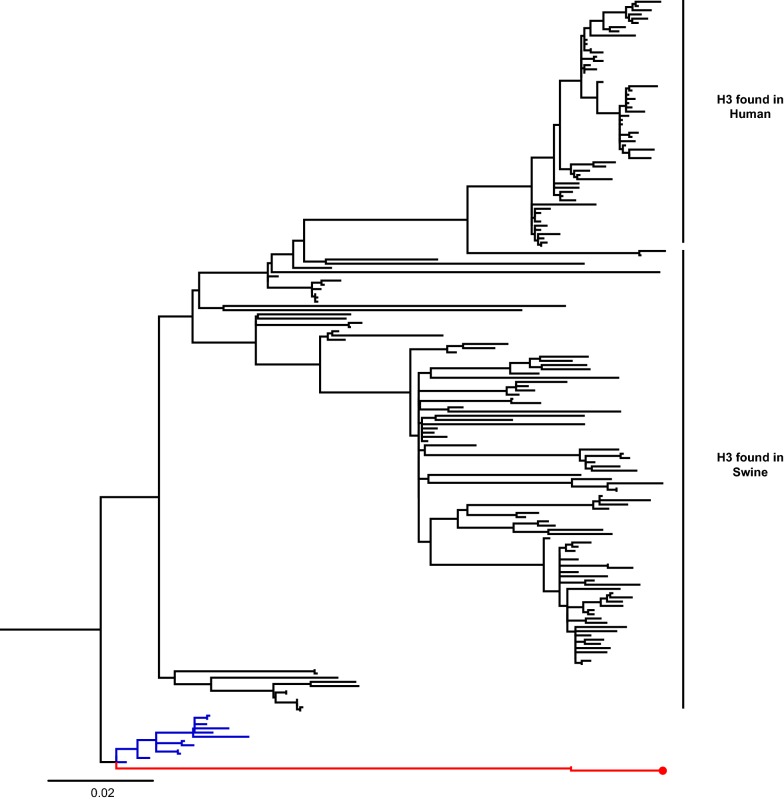
**Phylogenetic trees based on HA sequences of subtype H3.** Maximum Likelihood method and General Time Reversible model with a variation rate among sites given by gamma distribution with invariant sites (GTR + G + I) were used. Human and swine reference sequences are in black. Branches of Chilean IAVs-S sequences are in red, and the node of the selected virus for guinea pig infection is highlighted with a red circle. The closest related sequences to the Chilean H3 IAV-S correspond to human sequences, which are highlighted in blue.

HA sequences were clustered within the lineages Chile H1 human I, Chile H1 human II, Pandemic—Chile 2 and Chile H3 human I, previously described by Nelson et al. [4]. We selected 1 virus per lineage (4 viruses in total): A/swine/Chile/VN1401-4/2014(H1N2), A/swine/Chile/VN1401-274/2014(H1N2), A/swine/Chile/VN1401-559/2014(H1N1), and A/swine/Chile/VN1401-1824/2015(H3N2), respectively. According to the H1 classification described by Anderson et al. [16], the strains A/swine/Chile/VN1401-4/2014(H1N2) and A/swine/Chile/VN1401-274/2014(H1N2) were classified within the human seasonal lineage 1B.2, while the strain A/swine/Chile/VN1401-559/2014(H1N1) was classified within the pdmH1N1 lineage (1A.3.3.2). Chilean H1 and H3 sequences were most closely related to human seasonal H1N1 and H3N2 IAVs, respectively, from the late 1980s and early 1990s. The H1N2 and H3N2 viruses were grouped in independent monophyletic clusters, genetically distant from IAVs identified in swine and humans globally (Figures 1, 2, Additional files 1 and 2).

Whole genome sequencing of selected IAVs-S was performed at the Sequencing Core Laboratory of the Center for Research on Influenza Pathogenesis (CRIP), Icahn School of Medicine at Mount Sinai, New York City, NY, USA. Briefly, all viral segments were amplified by a multisegment reverse transcription-PCR (M-RTPCR), following a previously described protocol [17]. Multiplexed libraries were prepared and sequenced on an Illumina HiSeq 2500 platform in a single-end 100 nt run format, and the viral genomes were assembled de novo. The neuraminidase (NA) segment was evaluated by the same phylogenetic analysis previously described for the HA segment. Internal gene sequences were submitted to BLAST to reveal the closest sequences for each segment.

The Chilean N2 sequences were most closely related to human seasonal H3N2 IAVs from the late 1980s and early 1990s. These sequences were grouped in independent monophyletic clusters, genetically divergent from IAVs described in other countries. The N2 sequences of H1N2 viruses were genetically distant (13%) from that of H3N2 virus (Additional file 3). On the other hand, the N1 segment was closely related to pdmH1N1 viruses (up to 98% identity). All viruses contained internal genes derived from the pdmH1N1. The GenBank accession numbers for these sequences are provided in Additional file 4.

The four selected viruses were propagated in MDCK cells, aliquoted and stored at −80 °C until used. Viruses were then titrated in triplicate by median tissue culture infective dose (TCID_50_) assay. Briefly, confluent MDCK cells in 96-well plates were washed three times with 150 μL of PBS containing 1 µg/mL of TPCK-treated trypsin and inoculated with 100 µL of ten-fold serial dilutions of each virus (4 replicates per virus dilution). The cells were incubated at 37 °C and observed for CPE daily for 5 days. The virus titer was expressed in TCID_50_/mL according to the Reed-Muench method [18]. Immediately before the inoculation of animals, each virus was diluted to 10^5^ TCID_50_/300 µL in PBS containing 100 units/mL penicillin G, 100 µg/mL streptomycin sulfate, 250 ng/mL amphotericin B and 0.3% bovine serum albumin (PBS-PSA-BSA). This titer was confirmed by back-titration (TCID_50_ assay).

Twenty-four Pirbright strain guinea pigs were obtained from the Instituto de Salud Pública de Chile. They were acclimated for a week on a 12 h light/dark cycle and were allowed access to food and water ad libitum. Animals were then randomly separated into four groups of six animals each, which were inoculated intranasally with 300 µL of PBS-PSA-BSA containing 10^5^ TCID_50_ of each selected virus. Prior to virus inoculation and sample collections, the guinea pigs were anesthetized intramuscularly with a mixture of ketamine (30 mg/kg) and xylazine (2 mg/kg) [9]. Nasal washings were collected from animals on 1, 3, 5, 7, 9, and 11 days post-inoculation (dpi) by instilling 1 mL of PBS-PSA-BSA into the nostrils. Samples were centrifuged for 8 min at 6000 × *g* and 4 °C and the supernatants were stored at −80 °C. Serum samples were obtained from each animal at 0 (prior to inoculation) and 11 dpi (euthanasia). Procedures were conducted in biosafety level-2 (BSL-2) conditions and were approved by Institutional Animal Care and Use Committees of the Universidad de Chile, under protocol number 02-2016.

An ELISA to detect antibodies against IAVs was performed on serum samples, according to the manufacturer’s instructions (IDEXX Influenza A Ab Test, IDEXX Laboratories, Inc., Westbrook, ME, USA). The virus titration of each nasal wash sample was calculated by the TCID_50_ assay described above. Viral titers were compared among animal groups in each sampling time, and area under the curve (AUC) was calculated to compare the total shedding among virus strains. Kruskal–Wallis test followed by Dunn’s multiple comparisons test was performed for these analyzes, using the RStudio software (version 1.1.383). Statistical analysis of infection curves was performed by Mantel-Cox test, using GraphPad Prism 5 software (version 5.01). A *P* value of < 0.05 was considered statistically significant.

**Figure 3:**
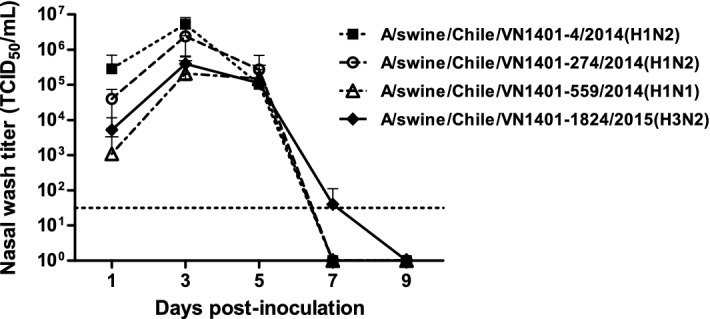
**Replication kinetics of novel reassortant IAVs-S in the guinea pig model.** Groups of 6 guinea pigs were intranasally inoculated with 10^5^ TCID_50_ of either A/swine/Chile/VN1401-4/2014(H1N2), A/swine/Chile/VN1401-274/2014(H1N2), A/swine/Chile/VN1401-559/2014(H1N1) or A/swine/Chile/VN1401-1824/2015(H3N2) viruses. Nasal wash samples were collected on 1, 3, 5, 7, and 9 dpi, and viral titers were determined by TCID_50_ assay. Data are shown as mean ± SD. Horizontal dashed line represents the limit of detection of the assay (10^1.5^ TCID_50_/mL).

**Figure 4:**
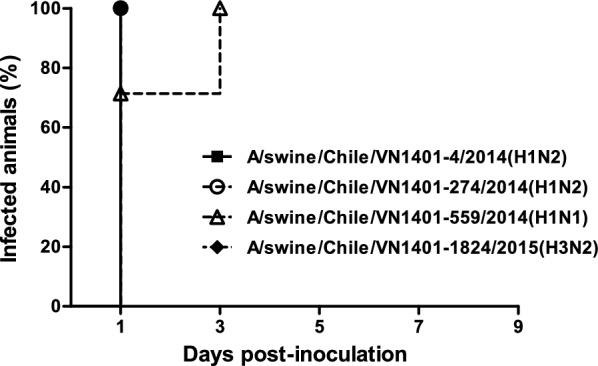
**Infection dynamics of novel reassortant IAVs-S in the guinea pig model.** Infection curves indicate de percent of animals that were found to be positive by TCID_50_ assay after inoculation with either A/swine/Chile/VN1401-4/2014(H1N2), A/swine/Chile/VN1401-274/2014(H1N2), A/swine/Chile/VN1401-559/2014(H1N1) or A/swine/Chile/VN1401-1824/2015(H3N2) viruses. Viral infection was determined on six guinea pigs per group on 1, 3, 5, 7, and 9 dpi.

Guinea pigs were seronegative to IAV at 0 dpi (Additional file 5). All viruses were able to infect guinea pigs, reaching a peak at 3 dpi with an average nasal viral load of 10^6.7^ TCID_50_/mL for A/swine/Chile/VN1401-4/2014(H1N2), 10^6.4^ TCID_50_/mL for A/swine/Chile/VN1401-274/2014(H1N2), 10^5.3^ TCID_50_/mL for A/swine/Chile/VN1401-559/2014(H1N1) and 10^5.6^ TCID_50_/mL for A/swine/Chile/VN1401-1824/2015(H3N2). The strains A/swine/Chile/VN1401-4/2014(H1N2) and A/swine/Chile/VN1401-274/2014(H1N2) showed higher viral titers than strains A/swine/Chile/VN1401-559/2014(H1N1) and A/swine/Chile/VN1401-1824/2015(H3N2) on 1 and 3 dpi (*P* < 0.05). The overall shedding during the experiment was statistically higher (*P* < 0.05) in animals infected with strains A/swine/Chile/VN1401-4/2014(H1N2) and A/swine/Chile/VN1401-274/2014(H1N2) than those infected with strains A/swine/Chile/VN1401-559/2014(H1N1) and A/swine/Chile/VN1401-1824/2015(H3N2) (Figure 3). Moreover, 1 of 6 animals inoculated with the pdmH1N1 virus did not show viral titers at 1 dpi. However, there was no statistical difference in the infection rate among the different groups (*P* = 0.94) (Figure 4).

The seroconversion rates, determined by ELISA at 11 dpi, were higher in animals inoculated with A/swine/Chile/VN1401-4/2014(H1N2) (6 out of 6) and A/swine/Chile/VN1401-274/2014(H1N2) (4 out of 6) than those inoculated with A/swine/Chile/VN1401-559/2014(H1N1) (2 out of 6) and A/swine/Chile/VN1401-1824/2015(H3N2) (2 out of 6) (Additional file 5). Note that all animals were infected, and that the ELISA test was only performed to assess the percentage of early seroconverted animals at the end of the experiment (11 dpi), since at that time point it is premature to determine overall seroconversion [19].

The viral load decreased by 5 dpi, and most of the animals became negative by 7 dpi, except for 2 animals infected with the A/swine/Chile/VN1401-1824/2015(H3N2) virus. The viral clearance was observed in all groups between 7 and 9 dpi (Figure 3).

## Discussion

These results demonstrate that novel reassortant H1N2 and H3N2 IAVs-S with pandemic internal genes were able to infect, replicate and be shed efficiently in the upper respiratory tract of guinea pigs without prior adaptation. These viruses were compared with a pdmH1N1 virus, lineage that has been well studied in the guinea pig model since its emergence in 2009 [7, 8, 20]. Overall, all viruses had similar shedding kinetics except that both H1N2 viruses showed higher shedding titers than pdmH1N1 and H3N2 viruses. On the other hand, only animals inoculated with the H3N2 virus showed titers at 7 dpi. Using guinea pig model, Sun et al. [21] reported similar shedding kinetics for H1N2 and H3N2 IAVs-S isolated in 2006 in China and a pdmH1N1 virus isolated from human in 2009, where H3N2 IAVs-S took a longer time to clear from the nasopharyngeal cavity than pdmH1N1 and seasonal human viruses.

This is of importance, since the respiratory system of guinea pigs has anatomical and physiological characteristics comparable to those of humans [11] along with a similar IAV receptor distribution [21]. Hence, these results suggest that these novel reassortant IAVs-S might be capable of replicating efficiently in other mammal hosts, such as humans.

Some cases of zoonotic human infections with swine-origin H1N2 and H3N2 variants, which contain a pandemic M gene, have been reported in North America [22, 23]. The pandemic M gene has been hypothesized as a critical factor in these zoonotic events, because it is thought to contribute to increased IAV transmission [20]. In addition to the pandemic internal genes, the H1N2 and H3N2 IAVs-S reported here also have human-origin HA genes [4], which may lead to an increase in the risk of human-to-human transmission if they are introduced back into humans. Recently, a pig farm worker became infected with a reassortant H1N2 IAV-S in Brazil (lineage 1B.2.2). This virus also had a human-origin HA (introduced into swine in the late 1990s) and pandemic internal genes [24]. Notably, H1N2 viruses have been frequently detected in Chilean commercial swine farms [4], underscoring the risk of this strain as a potential zoonotic virus.

Recently a Chilean H1N2 strain was detected in a backyard farm in 2014 [25], which unexpectedly had 100% of nucleotide identity with the HA and internal genes of A/swine/Chile/VN1401-4/2014(H1N2) strain used in this study. This lineage has frequently been found in commercial swine farms since 2012 [4]. Hence, one plausible explanation is that the backyard farm virus could be a result of a spillover event from a commercial farm, a situation that has been reported in Chile for other swine viruses such as porcine reproductive and respiratory syndrome virus [26].

There exist other animal models for IAV research, such as mouse and ferret. The main drawback to the mouse model is the need to use mouse-adapted viruses in order to achieve productive infection [6]. Bravo-Vasquez et al. [25] obtained a lower average nasal titer (10^3.5^ TCID_50_ approx.) at 3 dpi in mice compared to the nasal titer obtained at the same time point in this study (10^6.7^ TCID_50_), using the same inoculation dose (10^5^ TCID_50_) and a similar Chilean H1N2 IAV-S strain. Moreover, anatomical features of mouse airways are different from that of humans [11], and sialic acid α2,6-Gal receptor is rarely detected in the upper respiratory tract of BALB/c mice [21]. On the other hand, ferret is a validated model that is susceptible to influenza infection and develops symptoms similar to those of humans [27]. Nonetheless, the high cost and lack of commercial availability of ferrets in countries like Chile is a great disadvantage for the use of these animals. Although one limitation of the guinea pig model is the lack of clinical signs due to influenza infection [28], it is a valuable model for assessing viral replication, shedding and transmission.

Overall, these results highlight the guinea pig model as a useful tool to assess novel IAVs-S circulating in swine. Recently, other H1 IAVs-S classified within the human seasonal lineage 1B.2 have also been identified in Argentina, Brazil, China, Mexico, and Vietnam, highlighting the need to characterize these viruses more thoroughly [16]. Hence, guinea pigs could be used to further characterize these IAVs-S in challenge and transmission studies, to evaluate their zoonotic potential, and for the development and updating of IAV-S vaccines. Furthermore, studies related to genetic diversity and distribution of IAV in swine and human population in Chile and the region are necessary to fully evaluate the zoonotic potential of these viruses.

## Additional files

**Additional file 1.** **Phylogenetic trees based on HA sequences of subtype H1.** Maximum Likelihood method and General Time Reversible model with a variation rate among sites given by gamma distribution with invariant sites (GTR + G + I) were used. Human and swine reference sequences are in black. Chilean IAVs-S sequences are in red, and nodes of selected viruses for guinea pig infection are highlighted with a red circle.

**Additional file 2.** **Phylogenetic trees based on HA sequences of subtype H3.** Maximum Likelihood method and General Time Reversible model with a variation rate among sites given by gamma distribution with invariant sites (GTR + G + I) were used. Human and swine reference sequences are in black. Chilean IAVs-S sequences are in red, and the node of the selected virus for guinea pig infection is highlighted with a red circle. The closest related sequences to the Chilean H3 IAV-S correspond to human sequences, which are highlighted in blue.

**Additional file 3.** **Phylogenetic tree based on NA sequences of subtype N2.** Trees were inferred using the Maximum Likelihood method and a General Time Reversible model with a variation rate among sites given by gamma distribution with invariant sites (GTR + G + I). Human and swine reference sequences are represented in black. Chilean IAVs-S used for guinea pig infection sequences are shown in red.

**Additional file 4.** **GenBank accession numbers of all sequenced gene segments of the IAVs-S described in this study.**

**Additional file 5.** **Seroconversion of guinea pigs infected with novel reassortant IAVs-S.** Six guinea pigs were intranasally inoculated with either (A) A/swine/Chile/VN1401-4/2014(H1N2), (B) A/swine/Chile/VN1401-274/2014(H1N2), (C) A/swine/Chile/VN1401-559/2014(H1N1) or (D) A/swine/Chile/VN1401-1824/2015(H3N2) viruses. An NP competitive ELISA test was performed with serum samples obtained from each animal at 0 (prior inoculation) and 11 dpi (euthanasia). Results are expressed as the sample to negative control (S/N) ratio from optical density of each sample. Horizontal dashed line represents the threshold value, where S/N ratios < 0.6 were considered positive.

## Abbreviations

CPE: cytopathic effect
HA: hemagglutinin
IAV: influenza A virus
M: matrix
MDCK: Madin-Darby Canine Kidney
PBS: phosphate-buffered saline
PBS-PSA-BSA: PBS containing 100 units/mL penicillin G, 100 µg/mL streptomycin sulfate, 250 ng/mL amphotericin B and 0.3% bovine serum albumin
pdmH1N1: pandemic H1N1
TCID_50_: median tissue culture infective dose
TPCK: *N*-tosyl-l-phenylalanyl chloromethyl ketone

## References

[1] Nelson MI, Wentworth DE, Culhane MR, Vincent AL, Viboud C, LaPointe MP, Lin X, Holmes EC, Detmer SE (2014). Introductions and evolution of human-origin seasonal influenza A viruses in multinational swine populations. J Virol.

[2] Nelson MI, Viboud C, Vincent AL, Culhane MR, Detmer SE, Wentworth DE, Rambaut A, Suchard MA, Holmes EC, Lemey P (2015). Global migration of influenza A viruses in swine. Nat Commun.

[3] Vincent A, Awada L, Brown I, Chen H, Claes F, Dauphin G, Donis R, Culhane M, Hamilton K, Lewis N, Mumford E, Nguyen T, Parchariyanon S, Pasick J, Pavade G, Pereda A, Peiris M, Saito T, Swenson S, Van Reeth K, Webby R, Wong F, Ciacci-Zanella J (2014). Review of influenza A virus in swine worldwide: a call for increased surveillance and research. Zoonoses Public Health.

[4] Nelson M, Culhane MR, Rovira A, Torremorell M, Guerrero P, Norambuena J (2015). Novel human-like influenza A viruses circulate in swine in Mexico and Chile. PLoS Curr.

[5] Steel J, Staeheli P, Mubareka S, García-Sastre A, Palese P, Lowen AC (2010). Transmission of pandemic H1N1 influenza virus and impact of prior exposure to seasonal strains or interferon treatment. J Virol.

[6] Thangavel RR, Bouvier NM (2014). Animal models for influenza virus pathogenesis, transmission, and immunology. J Immunol Methods.

[7] Zhang H, Li X, Ma R, Li X, Zhou Y, Dong H, Li X, Li Q, Zhang M, Liu Z, Wei B, Cui M, Wang H, Gao J, Yang H, Hou P, Miao Z, Chai T (2013). Airborne spread and infection of a novel swine-origin influenza A (H1N1) virus. Virol J.

[8] Wiersma LC, Vogelzang-van Trierum SE, van Amerongen G, van Run P, Nieuwkoop NJ, Ladwig M, Banneke S, Schaefer H, Kuiken T, Fouchier RA, Osterhaus AD, Rimmelzwaan GF (2015). Pathogenesis of infection with 2009 pandemic H1N1 influenza virus in isogenic guinea pigs after intranasal or intratracheal inoculation. Am J Pathol.

[9] Lowen AC, Mubareka S, Tumpey TM, García-Sastre A, Palese P (2006). The guinea pig as a transmission model for human influenza viruses. Proc Natl Acad Sci U S A.

[10] Tang X, Chong KT (2009). Histopathology and growth kinetics of influenza viruses (H1N1 and H3N2) in the upper and lower airways of guinea pigs. J Gen Virol.

[11] Canning BJ, Chou Y (2008). Using guinea pigs in studies relevant to asthma and COPD. Pulm Pharmacol Ther.

[12] World Health Organization. http://www.who.int/csr/resources/publications/swineflu/realtimeptpcr/en/. Accessed 17 May 2018

[13] GenBank. https://www.ncbi.nlm.nih.gov/genbank/. Accessed 10 Apr 2018

[14] Basic Local Alignment Search Tool (BLAST). https://blast.ncbi.nlm.nih.gov/Blast.cgi. Accessed 10 Apr 2018

[15] Influenza Research Database (IRD). https://www.fludb.org/brc/home.spg?decorator=influenza. Accessed 10 Apr 2018

[16] Anderson TK, Macken CA, Lewis NS, Scheuermann RH, Reeth KV, Brown IH, Swenson SL, Simon G, Saito T, Berhane Y, Ciacci-Zanella J, Pereda A, Davis CT, Donis RO, Webby RJ, Vincent AL (2016). A phylogeny-based global nomenclature system and automated annotation tool for H1 hemagglutinin genes from swine influenza A viruses. mSphere.

[17] Zhou B, Donnelly ME, Scholes DT, St George K, Hatta M, Kawaoka Y, Wentworth DE (2009). Single-reaction genomic amplification accelerates sequencing and vaccine production for classical and Swine origin human influenza A viruses. J Virol.

[18] Reed LJ, Muench H (1938). A simple method of estimating fifty per cent endpoints. Am J Epidemiol.

[19] Wang Z, Yang H, Chen Y, Tao S, Liu L, Kong H, Ma S, Meng F, Suzuki Y, Qiao C, Chen H (2017). A single-amino-acid substitution at position 225 in HA alters the transmissibility of Eurasian avian-like H1N1 swine influenza virus in guinea pigs. J Virol.

[20] Chou YY, Albrecht RA, Pica N, Lowen AC, Richt JA, García-Sastre A, Palese P, Hai R (2011). The M segment of the 2009 new pandemic H1N1 influenza virus is critical for its high transmission efficiency in the guinea pig model. J Virol.

[21] Sun Y, Bi Y, Pu J, Hu Y, Wang J, Gao H, Liu L, Xu Q, Tan Y, Liu M, Guo X, Yang H, Liu J (2010). Guinea pig model for evaluating the potential public health risk of swine and avian influenza viruses. PLoS One.

[22] Centers for Disease Control and Prevention (CDC). https://www.cdc.gov/flu/spotlights/h1n2v-cases-mn.htm. Accessed 10 Apr 2018

[23] Epperson S, Jhung M, Richards S, Quinlisk P, Ball L, Moll M, Boulton R, Haddy L, Biggerstaff M, Brammer L, Trock S, Burns E, Gomez T, Wong KK, Katz J, Lindstrom S, Klimov A, Bresee JS, Jernigan DB, Cox N, Finelli L, Influenza A (H3N2)v Virus Investigation Team (2013). Human infections with influenza A(H3N2) variant virus in the United States, 2011-2012. Clin Infect Dis.

[24] Resende PC, Born PS, Matos AR, Motta FC, Caetano BC, Debur MD, Riediger IN, Brown D, Siqueira MM (2017). Whole-genome characterization of a novel human influenza A(H1N2) virus variant, Brazil. Emerg Infect Dis.

[25] Bravo-Vasquez N, Karlsson EA, Jimenez-Bluhm P, Meliopoulos V, Kaplan B, Marvin S, Cortez V, Freiden P, Beck MA, Hamilton-West C, Schultz-Cherry S (2017). Swine influenza virus (H1N2) characterization and transmission in ferrets, Chile. Emerg Infect Dis.

[26] Neira V, Brito B, Mena J, Culhane M, Apel MI, Max V, Perez P, Moreno V, Mathieu C, Johow M, Badia C, Torremorell M, Medina R, Ortega R (2017). Epidemiological investigations of the introduction of porcine reproductive and respiratory syndrome virus in Chile, 2013–2015. PLoS ONE.

[27] Belser JA, Katz JM, Tumpey TM (2011). The ferret as a model organism to study influenza A virus infection. Dis Model Mech.

[28] Bouvier NM, Lowen AC (2010). Animal models for influenza virus pathogenesis and transmission. Viruses.

