# Supplementary figures and images for: Infection of novel reassortant H1N2 and H3N2 swine influenza A viruses in the guinea pig model
Source: Vet Res. 2018 Jul 27;49:73. doi: 10.1186/s13567-018-0572-4 (PMC6062863; doi:10.1186/s13567-018-0572-4)

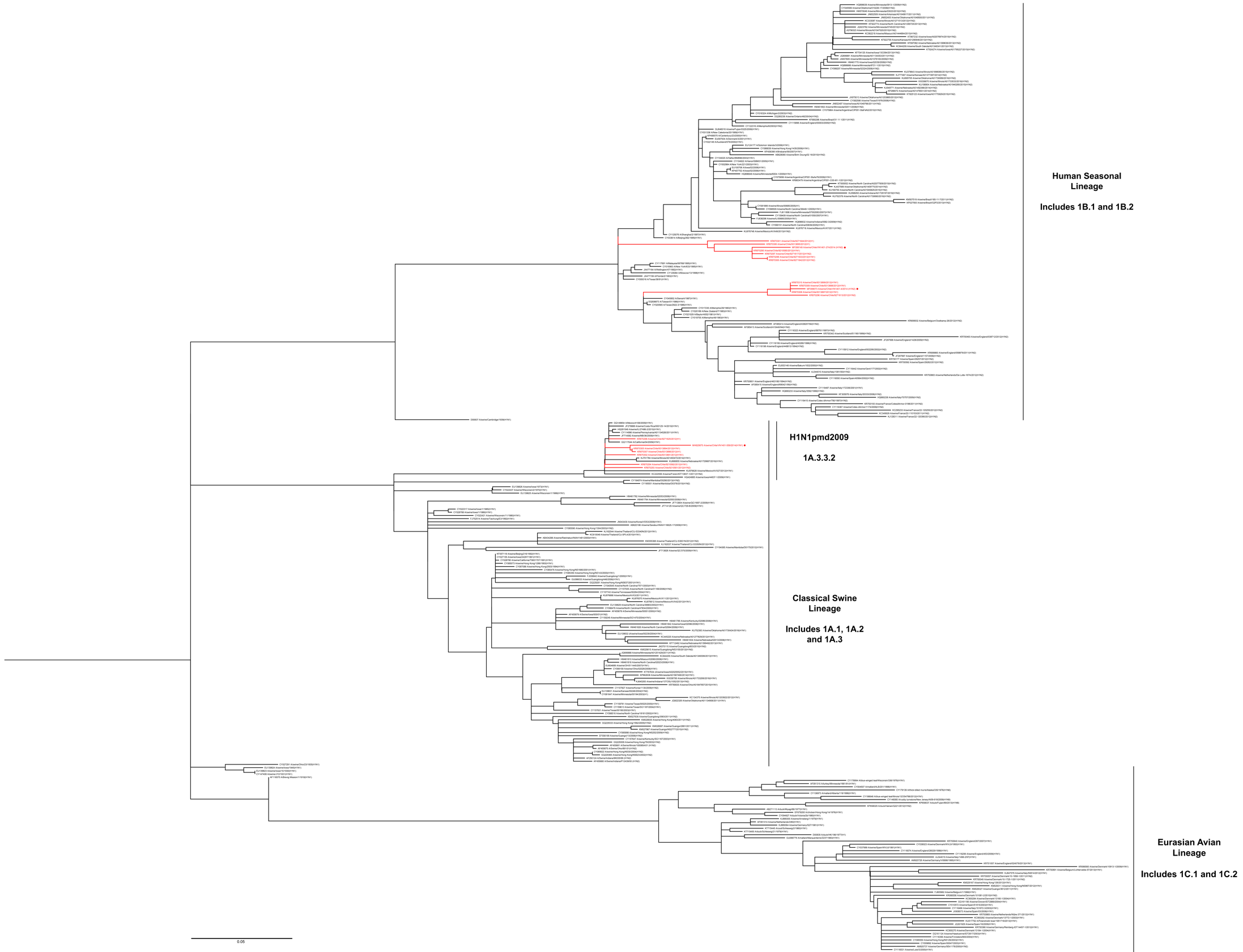

Supplement: Supplementary file 1 — Additional file 1. Phylogenetic trees based on HA sequences of subtype H1. Maximum Likelihood method and General Time Reversible model with a variation rate among sites given by gamma distribution with invariant sites (GTR + G + I) were used. Human and swine reference sequences are in black. Chilean IAVs-S sequences are in red, and nodes of selected viruses for guinea pig infection are highlighted with a red circle. [file 13567_2018_572_MOESM1_ESM.pdf]

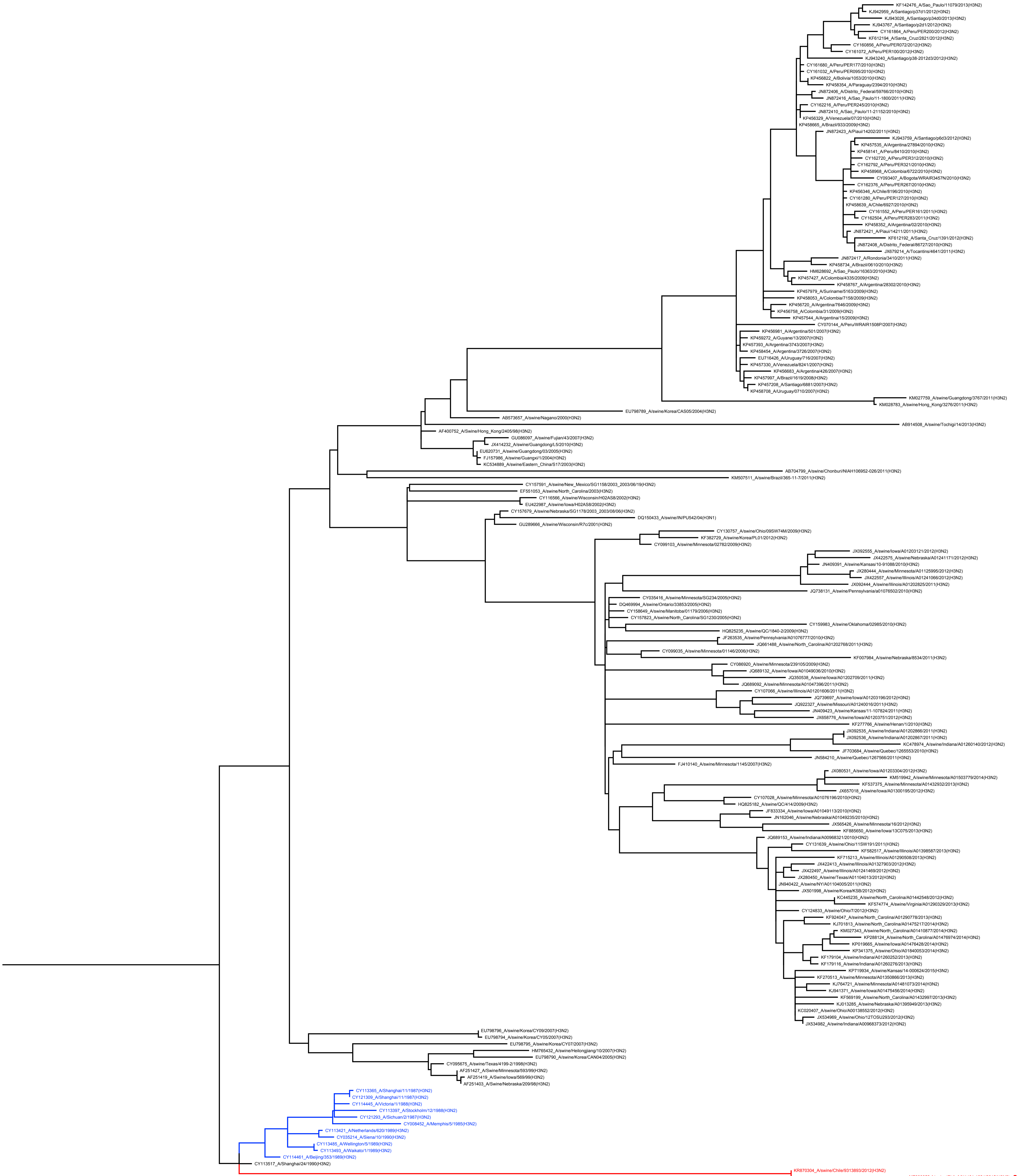

H3 found in Human

H3 found in Swine

Supplement: Supplementary file 2 — Additional file 2. Phylogenetic trees based on HA sequences of subtype H3. Maximum Likelihood method and General Time Reversible model with a variation rate among sites given by gamma distribution with invariant sites (GTR + G + I) were used. Human and swine reference sequences are in black. Chilean IAVs-S sequences are in red, and the node of the selected virus for guinea pig infection is highlighted with a red circle. The closest related sequences to the Chilean H3 IAV-S correspond to human sequences, which are highlighted in blue. [file 13567_2018_572_MOESM2_ESM.pdf]

# Human N2 (2000-2017)

# Swine N2

# Human N2 (1989-1999)

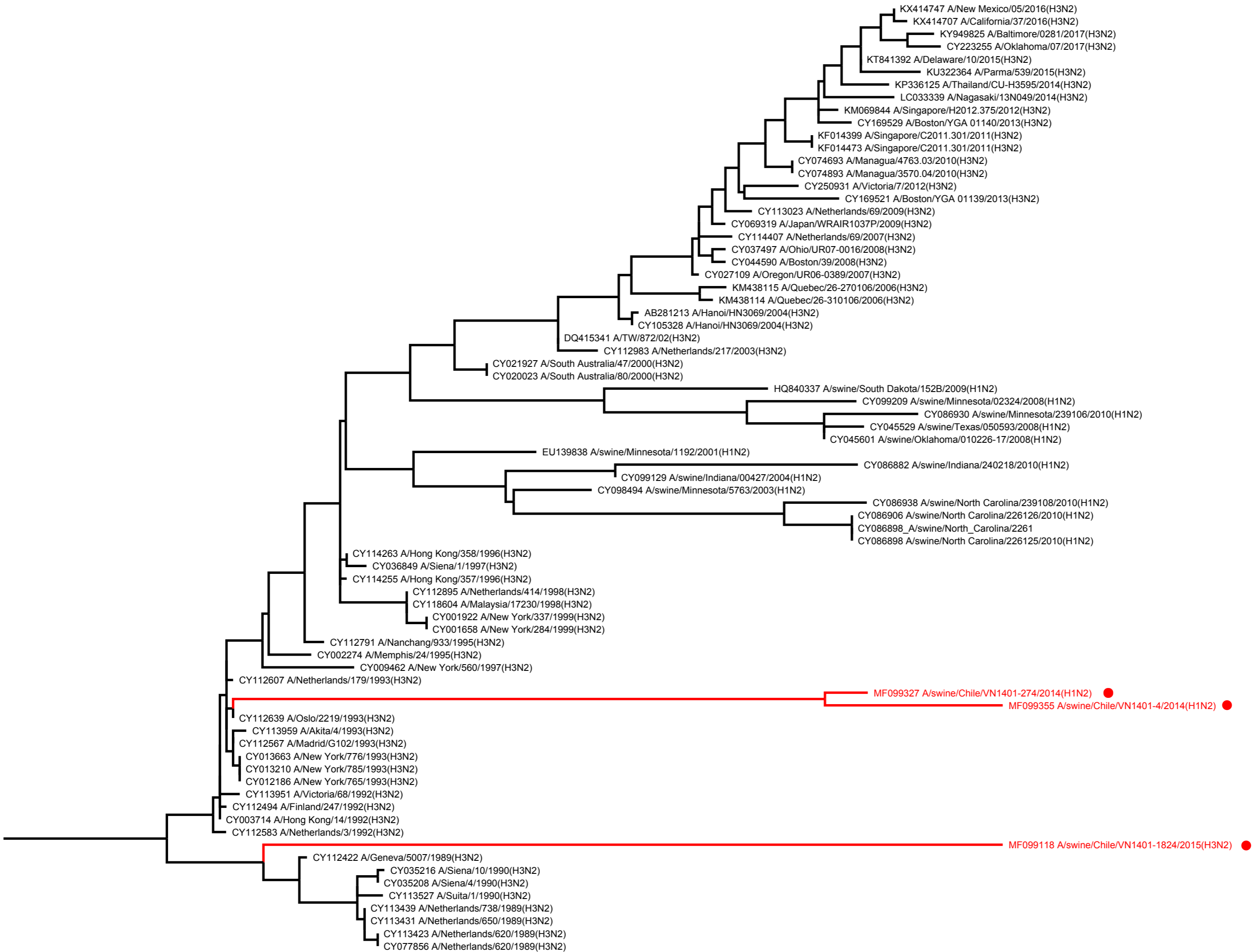

Supplement: Supplementary file 3 — Additional file 3. Phylogenetic tree based on NA sequences of subtype N2. Trees were inferred using the Maximum Likelihood method and a General Time Reversible model with a variation rate among sites given by gamma distribution with invariant sites (GTR + G + I). Human and swine reference sequences are represented in black. Chilean IAVs-S used for guinea pig infection sequences are shown in red. [file 13567_2018_572_MOESM3_ESM.pdf]

**A**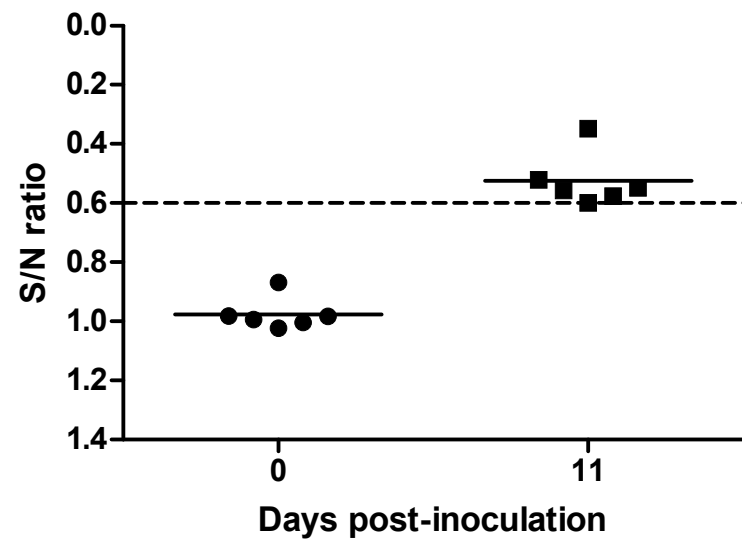**B**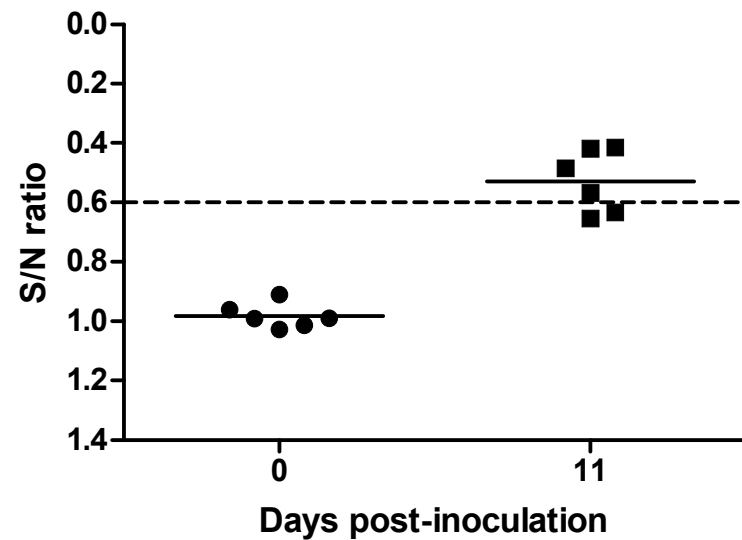**C**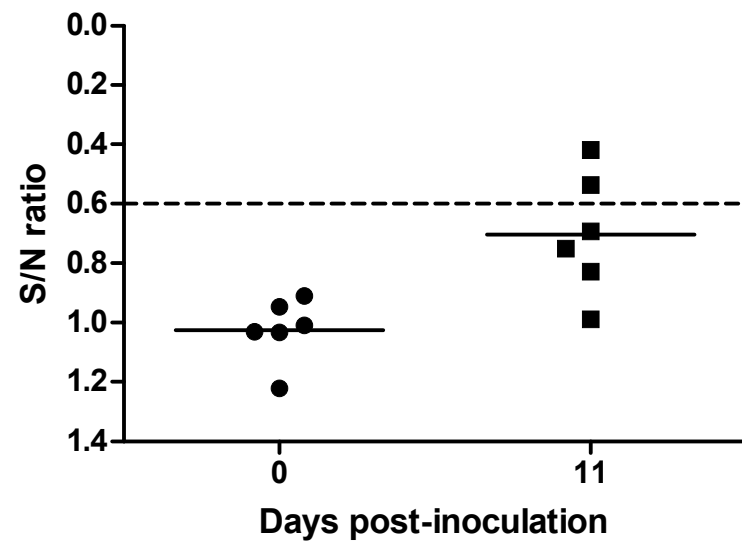**D**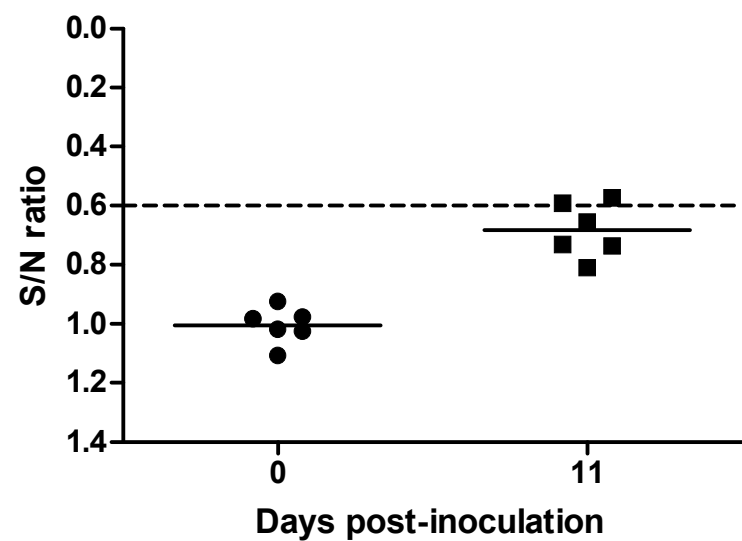

Supplement: Supplementary file 5 — Additional file 5. Seroconversion of guinea pigs infected with novel reassortant IAVs-S. Six guinea pigs were intranasally inoculated with either (A) A/swine/Chile/VN1401-4/2014(H1N2), (B) A/swine/Chile/VN1401-274/2014(H1N2), (C) A/swine/Chile/VN1401-559/2014(H1N1) or (D) A/swine/Chile/VN1401-1824/2015(H3N2) viruses. An NP competitive ELISA test was performed with serum samples obtained from each animal at 0 (prior inoculation) and 11 dpi (euthanasia). Results are expressed as the sample to negative control (S/N) ratio from optical density of each sample. Horizontal dashed line represents the threshold value, where S/N ratios < 0.6 were considered positive. [file 13567_2018_572_MOESM5_ESM.pdf]
